# Functional Status among Polymedicated Geriatric Inpatients at Discharge: A Population-Based Hospital Register Analysis

**DOI:** 10.3390/geriatrics6030086

**Published:** 2021-09-03

**Authors:** Filipa Pereira, Boris Wernli, Armin von Gunten, María del Rio Carral, Maria Manuela Martins, Henk Verloo

**Affiliations:** 1 Abel Salazar Institute of Biomedical Sciences, University of Porto, 4050-313 Porto, Portugal; 2 School of Health Sciences, HES-SO Valais/Wallis, 1950 Sion, Switzerland; henk.verloo@hevs.ch; 3 Swiss Centre of Expertise in the Social Sciences (FORS), University of Lausanne, 1015 Lausanne, Switzerland; boris.wernli@fors.unil.ch; 4 Service of Old Age Psychiatry, Lausanne University Hospital, 1011 Lausanne, Switzerland; armin.von-gunten@chuv.ch; 5 Research Center for Psychology of Health, Aging and Sport Examination, Institute of Psychology, University of Lausanne, 1015 Lausanne, Switzerland; maria.delriocarral@unil.ch; 6 NursID, CINTESIS, Higher School of Nursing of Porto, 4050-040 Porto, Portugal; mmartins@esenf.pt

**Keywords:** polypharmacy, older adults, hospital discharge, hospital register, population-based, functional status, physical impairment, cognitive disorders, epidemiology

## Abstract

This study explored and compared the functional status of polymedicated and non-polymedicated geriatric inpatients at hospital discharge. We used a cross-sectional registry of geriatric patients’ hospital records from a multi-site public hospital center in Switzerland. The analysis included all inpatients aged 65 years old or more admitted between 1 January 2015 and 31 December 2017 (n = 53,690), of whom 67.5% were polymedicated at hospital discharge, 52.1% were women (n = 18,909), and 42.7% were 75–84 years old (n = 15,485). On average, the polymedicated patients’ hospital lengths of stay were six days longer, they presented with more than three comorbidities, and they were prescribed more than nine medications at hospital discharge (*p* < 0.001). They showed more frequent general mobility decline (43.2% vs. 41.9%), gait disorders (46.2% vs. 43%), fatigue (48.6% vs. 43.4%) and dependence on lower-body care (49.7% vs. 47.6%), and presented a higher malnutrition risk (OR = 1.411; 95%CI 1.263–1.577; *p* < 0.001). However, the non-polymedicated inpatients had proportionally more physical and cognitive impairments. The comparison of the functional status of polymedicated and non-polymedicated geriatric inpatients at hospital discharge is important for clinicians trying to identify and monitor those who are most vulnerable to functional decline, and to design targeted strategies for the prevention of functional impairment and related adverse health outcomes.

## 1. Introduction

The world population is rapidly ageing. As people everywhere live longer, there will be dramatic increases in the prevalence of many chronic conditions—due to multiple underlying factors—that tend to occur later in life [1]. Older people living with chronic conditions often present with a functional decline that leads to them becoming significant users of healthcare services, particularly acute care [2,3]. Between 2010 and 2015, hospitalizations in Switzerland of people aged 60 years old or more increased by 11.6% for men and 9.0% for women [4]. This is particularly worrying because hospital admissions are frequently associated with functional decline, a greater risk of readmissions, and unplanned institutionalization in long-term care facilities [5,6,7]. Hospitalizations, especially if repeated and prolonged, may produce adverse events and irreversible health outcomes, precipitating geriatric syndromes, functional impairment, institutionalization, and even early death [8,9,10]. Older inpatients are at a greater risk of declining functional status, frailty, delirium, falls, medication toxicity, nosocomial infections, malnutrition, dehydration, immobilization, and pressure sores while hospitalized [11].

Functional status is commonly defined as the basic physical and cognitive abilities enabling the routine activities of daily living—including eating, bathing, dressing, transferring, and toileting—and the everyday duties required to live in one’s own home, especially if one lives alone [12]. According to Inouye’s unifying conceptual model [13], shared risk factors among older adults—including older age and functional decline—may lead to geriatric syndromes, which may, in turn, lead to the overarching geriatric syndrome of frailty. Both generate feedback mechanisms intensifying those shared risk factors and geriatric syndromes [13]. Thus, geriatric inpatients are susceptible to complications and adverse events which are not directly related to the illness or event for which they were hospitalized [8,10,14,15].

The major increases in life expectancy have largely been due to advances in healthcare, science and technology, notably the optimization and personalization of medication, enabling people to cope with the complex manifestations of their chronic conditions and their impacts on their daily lives [16]. Hence, the use of multiple medications, usually referred to as polypharmacy (PP), is common in older multimorbid populations, as one or more medications may be used to treat each health condition [17]. Although there is no consensus on the number of medications that should be considered the threshold for PP, a systematic review of PP definitions by Masnoon et al. found that the most commonly used numerical definition in research was five or more medications daily [18]. However, due to age-related pharmacokinetic and pharmacodynamic changes, PP places older people at a greater risk of medication-related problems (MRPs) such as adverse medication reactions, medication errors and potentially inappropriate prescriptions [19,20]. MRPs can engender functional decline and exacerbate medical conditions [21,22]. Consequently, MRPs are a frequent cause of readmission and avoidable health costs [23,24].

Midão and Giardini [25] examined the prevalence of PP and its associated effects among 29,342 older adults across 17 European countries and Israel, finding a range of PP from 26.3% to 39.9%. The overall prevalence of PP among older adults from 65–74 years old was 26.7%, whereas it was 43.1% for those aged 85 or more. PP was also more prevalent among women (33.0%) than men (31.2%). As expected, their study revealed that older adults with fewer than two chronic diseases were less susceptible to PP—12.1% instead of 42.3% among those with two or more chronic diseases—and that the older adults who received more prescriptions were those with a poorer health status. A prospective three-year Finnish cohort study of 294 older adults aged 75 or more at the baseline showed that excessive PP—defined as the use of ten or more medications—was associated with an increased risk of malnutrition (*p* < 0.001), functional decline (*p* < 0.001) and cognitive impairment (*p* < 0.001) compared to non-polymedicated subjects [26]. Figueiredo and Teixeira [27] reported different predictors of difficulties in medication intake across 16 European countries. Their findings suggested that younger age (OR = 0.441; 95%CI 0.376–0.517), maintained physical ability (OR = 0.822; 95%CI 0.736–0.918), having a sense of meaning in life (OR = 0.781; 95%CI 0.636–0.960), and memory and concentration (OR = 0.822; 95%CI 0.736–0.918) were significant predictors of fewer difficulties in medication intake and lower risks of MRPs. One qualitative review also showed that vision, memory, swallowing and hearing can affect patients’ abilities to adhere to their medication prescriptions [28].

In addition to personal factors, nursing- and hospital-related factors may also contribute to functional decline [29]. Recovering from functional decline is challenging and can irreversibly impact older adults’ transitions of care and quality of life [30]. Prevention starts with recognizing which geriatric inpatients are at risk of functional decline [31]. Early rehabilitation protocols applied during acute hospitalization are crucial to the prevention of functional decline [32]. Furthermore, comprehensive geriatric assessments and nursing interventions focused on functional independence can reduce the risk of hospital-acquired functional decline [33].

Despite the complex, multi-dimensional clinical situations facing geriatric patients in acute care hospitals, most of the published studies used small cohort, cross-sectional sample designs to report on single causes of hospitalization and their complications [34,35,36]. To the best of our knowledge, no studies to date have compared the functional status at hospital discharge of polymedicated and non-polymedicated geriatric inpatients, aged 65 years old or more, using patient records extracted from a population-based hospital register. This study aimed to fill that gap.

We hypothesized that older inpatients presenting functional decline at hospital discharge have a greater risk of being polymedicated than those with a normal functional status.

## 2. Materials and Methods

### 2.1. Study Design, Setting and Study Population

A cross-sectional, registry-based study of geriatric patients’ hospital records was carried out. The data were derived from a large, longitudinal, electronic patient dataset extracted from the Valais Hospital (a public general hospital in the French-speaking part of Switzerland). Switzerland’s healthcare system combines public and private elements, with private health insurers operating in a highly regulated market, and with the State co-financing hospitals. The cantons cover 55% of inpatient treatment costs, and the health insurance companies cover 45%; for outpatient care, the health insurance companies cover 100% of the costs. The current trend is, therefore, to favor outpatient treatment, and cantons are trying to shorten the hospital length of stay as much as possible [37].

The hospital register’s electronic health records included all of the inpatients aged 18 years old or more admitted or readmitted between 1 January 2015 and 31 December 2017 (N = 105,243). Ethical approval (2018-02196) from the Human Research Ethics Committee of the Canton of Vaud (CER-VD) was obtained on 1 February 2019 and enabled the hospital’s data warehouse to provide us with the appropriate dataset. In accordance with the Swiss Federal Act on Personal Data Protection, regulated at the federal and cantonal levels [38], sensitive personal data in the patients’ records were excluded from the dataset, such as their date of birth, address, level of education and profession.

### 2.2. Customizing the Dataset and Selecting Variables of Interest

The extracted dataset was composed of sociodemographic, administrative, clinical, medical and medication data, and was delivered as an Excel csv. file. A three-year period was selected based on the availability of systematic, well-coded patient data, although no electronic patient records for the period were available from the adult psychiatry department. In order to fulfill the study’s aim, the dataset was cleaned to focus on geriatric inpatients aged 65 or more at hospital discharge. In the absence of a unique patient identifier, the number of different patients and their readmission rate could not be explored.

### 2.3. Study Variables Selected

The variables were selected according to the study’s aim and the shared risk factors for geriatric syndromes responsible for functional decline and adverse health outcomes among older adults, as documented in Inouye’s unifying conceptual model [13]. These shared risk factors are older age, cognitive impairment, functional impairment and impaired mobility, as identified through five common geriatric syndromes (pressure ulcers, incontinence, falls, functional decline and delirium) [13].

The analysis included the following variables for each hospitalization: (i) sociodemographic characteristics, including the year of birth, sex, pre-hospital provenance and discharge destination, or death during hospitalization, and hospital length of stay (LOS); (ii) physical status, including general mobility, mobility for changing position, gait, balance disorders, fall risk, fatigue, independence in upper and lower body care, upper and lower body dressing/undressing, eating and drinking, swallowing disorders, bladder and bowel continence, independence in urination and defecation, hearing, vision, acute and chronic pain, malnutrition risk and Braden risk (for the prediction of pressure sore risk [39]); (iii) cognitive status, including alertness/consciousness, orientation, concentration, verbal expression, capacity and skills to react to the demands of daily life, and the ability to learn; (iv) the number of comorbidities, calculated at each hospitalization using the 10th revision of the International Statistical Classification of Diseases and Related Health Problems (ICD-10) [40]; and (v) the number of medications taken at discharge, as well as medications that increased fall and/or delirium risk. PP was defined as five or more prescribed medications [18].

### 2.4. Data Analysis

After an inspection of the dataset for extraction errors, missing values, and data consistency by two experienced data managers (BW, HV), the data were imported into Statistical Package for the Social Sciences (SPSS) software, version 27.0 (IBM-SPSS Inc., Chicago, IL, USA), for analysis. The missing values were analyzed based on the best practices for longitudinal datasets [41]. In accordance with the study’s purpose, several categorical variables were recoded for the planned statistical analysis. The “discharge destination” variable, which had 16 categories in the original dataset, was recoded into home, deceased in hospital, and other destinations.

All of the physical and cognitive status variables were recoded as dichotomous variables (full ability/normal or impairment/disorder). The parametric properties were analyzed for the normality of their distributions and the equality of their variances using the Kolmogorov–Smirnov test. Non-parametric tests were performed for the variables with non-normal distributions in order to compare polymedicated and non-polymedicated geriatric inpatients. A descriptive analysis measured the frequencies for categorical variables and central tendencies for continuous and ordinal variables. The associations between polymedicated and non-polymedicated geriatric inpatients and their physical and cognitive conditions were computed using Cramér’s V. Multivariate logistic regressions were computed to explore the risks of PP at hospital discharge associated with functional decline. Boxplots were used to identify the outliers in the dataset. In order to increase the study’s internal validity, we adjusted our results for age, sex and LOS. The significance level was set at *p* < 0.01 in all of the analyses, given the large number of hospitalizations considered.

## 3. Results

### 3.1. Dataset

#### 3.1.1. Total and Retrieved Study Population

The total dataset was composed of 105,243 hospitalization records. After applying the inclusion criteria for older adult inpatients aged 65 or more, 53,690 hospitalizations (51% of the dataset) from 2015–2017 were included in our analysis (Table 1).

#### 3.1.2. Missing Values

There were few to no missing values per variable (99% of variables had less than 20% missing values, Supplementary Table S1), and these were missing at random. Bearing in mind the very low level of missing values, the large population size and our biostatistician’s recommendation (BW), we did not apply any missing value strategies, and mentioned the population of interest for each variable.

#### 3.1.3. Outliers

The hospital LOS variable featured some significantly long outliers, which were explored using boxplots (10% of hospitalizations). Based on the best practice, outliers of more than 200 days in hospital were replaced by the LOS mean score.

### 3.2. Sociodemographic Characteristics

Our population of interest was distributed into the age categories of 65–74, 75–84, and 85 and over, containing 18,882 (35.2%), 21,818 (40.6%) and 12,990 (24.2%) older adults, respectively. The mean age was 78.4 (SD = 7.9), the median was 78 (IQR = 72–84), and they ranged from 65–106 years old (Table 1), with 28,018 (52.2%) female geriatric inpatients. However, the geriatric inpatients aged from 65–74 were predominantly male. The proportion of women hospitalized increased from 75 years old, whereas for men, the proportion decreased significantly (*p* < 0.001).

Almost three quarters (n = 38,324; 71.4%) of the geriatric inpatients were admitted from their homes, whereas one quarter (n = 15,366; 28.6%) were transferred from other healthcare settings. A smaller number (n = 1002; 1.9%) were referred to the hospital from long-term care facilities such as nursing homes. However, only 62.9% of those geriatric inpatients were discharged back home, and 7.4% were discharged to long-term care facilities. In addition, 4.9% (n = 2646) of the hospitalizations resulted in death in the hospital. The LOS of the geriatric inpatients varied from 1 to 197 days, with an average of 12.1 days (SD = 14.5) and a median of 8 days (IQR = 4–15). As their age increased, the patients remained hospitalized for longer (Table 1).

Two thirds (67.5%) of the geriatric inpatients were polymedicated at hospital discharge. Most of the polymedicated geriatric inpatients were women (n = 18,909; 52.1%). The biggest group was aged from 75–84 years old (n = 15,485; 42.7%), and most had been admitted from (n = 24,740; 68.2%) and were returning to their homes (n = 23,747; 65.5%). Except for sex, all of the sociodemographic variables revealed statistically significant differences between the polymedicated and non-polymedicated geriatric inpatients (Table 1). On average, the polymedicated inpatients remained in hospital for six more days than the non-polymedicated inpatients (*p* < 0.001).

### 3.3. Description of Functional Status

#### 3.3.1. Physical Status

Most of the geriatric inpatients had their full physical abilities at hospital discharge (Table 2). However, more than 40% showed gait disorders (n = 21,768; 40.5%), fatigue (n = 22,638; 42.2%), or impairment in lower body care (n = 23,573; 43.9%) and dressing/undressing (n = 22,288; 41.5%). There were significant differences between the groups, with a *p* < 0.001, for all of the physical variables except balance disorders, impairment in lower body dressing/undressing, chronic pain, hearing and vision. Within-group analyses showed that the polymedicated inpatients had proportionally more physical impairments in general mobility (43.2% vs. 41.9% in non-polymedicated inpatients), gait (46.2% vs. 43%), fatigue (48.6% vs. 43.4%), and lower body care (49.7% vs. 47.6%). Polymedicated inpatients suffered more acute pain (38% vs. 35.8%) and presented with a greater malnutrition risk (83.7% vs. 72.7%). However, only 14,835 (27.6%) geriatric patients were assessed for malnutrition risk, and 11,995 (22.3%) had a moderate to high risk.

Non-polymedicated inpatients had proportionally more physical impairment than the polymedicated inpatients in terms of mobility for changing position (32.7% among non-polymedicated against 28.8% among polymedicated), upper body care (39.4% vs. 35.5%) and dressing/undressing (37.1% vs. 32.2%), eating (23.1% vs. 13.2%), drinking (17.1% vs. 8.1%), swallowing disorders (8.6% vs. 3.1%), bladder (33.1% vs. 31.5%) and bowel continence (21.2% vs. 14.6%), dependence in urination (30.4% vs. 26.0%) and defecation (27.0% vs. 22.0%), and a higher Braden risk (25.0% vs. 17.7%). The risk for falls was proportionally higher in the non-polymedicated group (20.4% vs. 12.3%).

#### 3.3.2. Cognitive Status

Most geriatric inpatients presented with normal cognitive status at discharge (Table 3). Nearly three quarters had preserved their sense of orientation (n = 37,800; 70.4%) and concentration (n = 42,105; 78.4%). The most well-preserved cognitive characteristics were alertness/consciousness (n = 45,779; 85.3%) and verbal expression (n = 43,572; 81.2%). In contrast, the least well-preserved cognitive abilities were the capacity and skills to react to the demands of daily life (n = 34,644; 64.5%) and the ability to learn (n = 34,125; 63.6%).

Within-group statistical analyses revealed that the proportions of geriatric inpatients with cognitive disorders were higher among the non-polymedicated for all of the cognitive variables (Table 3). More non-polymedicated geriatric inpatients were cognitively impaired than the polymedicated inpatients with regards to alertness/consciousness (12.0% vs. 1.7%) (*p* < 0.001). Indeed, the proportions were above 20% among non-polymedicated patients for orientation (26.3% of the non-polymedicated patients vs. 19.2% of the polymedicated patients), the capacity and skills to react to the demands of daily life (30.5% vs. 26.8%), and the ability to learn (31.5% vs. 27.8%). All of the cognitive indicators showed a significant difference between the groups (*p* < 0.001) (Table 3). Thus, geriatric inpatients with cognitive disorders were less medicated at hospital discharge. However, most patients whose cognitive status had not been assessed were non-polymedicated. Thus, the polymedicated geriatric inpatients were more often subject to clinical cognitive assessments.

### 3.4. Comorbidities and the Number of Medications Prescribed at Hospital Discharge

Significant differences were found between the polymedicated and non-polymedicated geriatric inpatients’ comorbidities (*p* < 0.001) (Supplementary Table S2). The median number of comorbidities was three higher among polymedicated (µ = 5.7; SD = 2.2; Med = 6; IQR = (4–8)) than among non-polymedicated patients (µ = 4.4; SD = 2.2; Med = 3; IQR = (3–6)). Polymedicated geriatric inpatients had a median of nine medications prescribed at hospital discharge (µ = 9.9; SD = 3.8; Med = 9; IQR = (7–12)) compared with just one for non-polymedicated ones (µ = 1.3; SD = 1.6; Med = 1; IQR = (0–3)), and one third had medications that increased their fall and/or delirium risk (n = 11,396; 32.8% among polymedicated inpatients vs. n = 3507; 26.6% among non-polymedicated; *p* < 0.001).

### 3.5. Associations between Polypharmacy and Functional Status

In order to investigate the strength of the relationships between PP and functional status at hospital discharge, we computed the symmetric Cramér’s V statistic. Based on Cohen’s classification of the strength of associations in healthcare studies [42,43], PP showed moderate associations (0.100–0.131) with fall risk and nutritional status (swallowing disorders, malnutrition risk, eating and drinking) (Table 4).

Regarding cognitive status, we found moderate associations between PP and concentration and verbal expression (0.118 and 0.127, respectively), and a strong association between PP and alertness/consciousness (0.221) (Table 5).

### 3.6. Multivariate Logistic Regressions

Multivariate logistic regressions were calculated to assess whether the moderate and strong associations between PP and physical and cognitive status significantly predicted PP when adjusted for sex, age and LOS. Table 6 presents odds ratios (ORs) suggesting that the odds of PP were greatest among geriatric inpatients with malnutrition (OR = 1.411; 95%CI 1.263–1.577; *p* < 0.001). In contrast, inpatients with swallowing disorders (OR = 0.702; 95%CI 0.574–0.858; *p* < 0.001), alertness/consciousness disorders (OR = 0.188; 95%CI 0.151–0.235; *p* < 0.001) and eating impairments (OR = 0.714; 95%CI 0.619–0.823; *p* < 0.001) were at less risk of PP. Adjusting the model for sex, age and LOS improved its fit, with log-likelihood values from 11,404 to 11,307, and a predictivity of PP, calculated using Nagelkerke’s R^2^, ranging from 0.139 to 0.150.

## 4. Discussion

Our principal findings revealed that the majority of geriatric inpatients were polymedicated at hospital discharge. This may reflect the current trend in Switzerland’s healthcare system of attempting to reduce the length of hospital stay as much as possible and promoting outpatient treatment instead of inpatient treatment. There is a possibility that this could lead to “medication-intensive hospital discharge”. In addition, polymedicated inpatients were likely to have three comorbidities and nine prescribed medications at discharge. They were more likely to be women, aged from 75–84 years old, living at home, and had longer hospital LOS. Although polymedicated inpatients showed proportionally more physical decline in general mobility, gait, fatigue, and lower body care, and presented with a higher malnutrition risk, our findings revealed that non-polymedicated inpatients had proportionally more physical and cognitive impairments, which partially refuted our initial hypothesis.

In our dataset, the older the patients were, the more medications they were prescribed. Our results are in line with a prospective cohort study in Italy by Nobili and Licata [44], in which the rate of PP at hospital discharge (67.0%) was also associated with age and comorbidities. However, this prevalence was substantially lower (26.3–39.9%) among other European older adults living in the community, according to Midão and Giardini [25]. Bearing in mind that PP exposes older adults to greater risks of MRPs [19,20,27], and that MRPs are a frequent cause of functional decline, hospital readmission, and avoidable health costs [23,24], prescribing so many medications to this vulnerable population at hospital discharge is paradoxical and should be a public health concern [45].

Our findings revealed an unsurprising association between the number of medications prescribed and the number of comorbidities. However, the pathophysiological mechanisms behind this association remain poorly defined. Although it is understandable (but not always justifiable) that the more different medical diagnoses a patient has, the more medications they are likely to be prescribed, the opposite—the impact of the number of medications on the number of diagnoses—is unclear, and needs to be disentangled in order to improve future policies on medication management for older adults.

In the present study, most of the polymedicated inpatients were women and aged 75–84 years old. Higher numbers of hospitalizations among women have been documented in several studies [25], and are explained by higher female life expectancy [46], a higher prevalence of frailty among women [47], women more frequently taking on the role of the informal caregiver [48], and different patterns of multimorbidity according to sex [49].

The differences between the LOS of polymedicated (µ = 13.8; SD = 15.1) and non-polymedicated (µ = 8.6; SD = 12.4) older adult inpatients were higher in our study than in Nobili’s (x = 10.2; SD = 8.6 and x = 10.9; SD = 7.7, respectively). Prolonged LOS is an indicator of both the clinical complexity of the hospitalized older adult’s case and the functional decline during that period triggering adverse events and irreversible health outcomes [8,44]. An optimal LOS is impossible to determine for older adults with multiple health conditions [50].

As expected, our findings highlighted that home-dwelling geriatric inpatients do not always return home after hospital discharge. This can be interpreted not only via the progression of the pathological processes leading to hospitalization but also through shared risk factors that lead to a downward spiral of accumulated adverse events and irreversible health outcomes in hospital [11,13]. In a previous study examining the same population, we explored institutionalization after hospitalization. Our findings showed that functional mobility impairment, dependency in the activities of daily living, and cognitive impairment were strong determinants of unplanned institutionalization (OR = 3.22, 95%CI 2.67–3.87; OR = 4.62, 95%CI 3.76–5.67; and OR = 3.75, 95%CI 3.06–4.59, respectively) [51]. These results complement another recent Swiss study by Tröster and Thalmann [52], which highlighted that frailty, physical decline and being underweight were significantly associated with a greater risk of subsequent institutionalization.

Based on the 23 physical variables considered in our study, 20 showed statistically significant differences between the two groups. Although non-polymedicated inpatients had more physical impairments across 13 variables, polymedicated inpatients had more physical impairments across seven variables. These results are inconsistent with the systematic review by Katsimpris and Linseisen [53], which reported strong bidirectional associations between PP and physical function outcomes among older adults. Our analyses are also different from those of the three-year prospective cohort study by Jyrkkä and Enlund [26], which showed that excessive PP was associated with declines in functional ability and cognitive capacity. The fact that non-polymedicated geriatric inpatients in our study presented proportionally greater levels of functional decline may be explained by the beneficial effects of certain medications on functional status. However, it may also be that physicians try not to prescribe complex treatments to physically and/or cognitively dependent older adults at their discharge, as they would probably struggle to manage them during this specific period. Therefore, our results may indicate growing concerns about prescriptive practices in Switzerland [54].

Moreover, our results revealed that the fall risk was proportionally higher in the non-polymedicated group, even though polymedicated geriatric inpatients had more medications increasing fall and/or delirium risk (n = 11,396; 32.8% vs. n = 3507; 26.6%) (*p* < 0.001). This contrasts with Zaninotto et al. [55], whose data from the English Longitudinal Study of Ageing revealed that the prevalence of people admitted to hospital due to a fall increased according to the number of medications taken, from 1.5% of people reporting no medications being admitted for a fall, to 7.9% of those with PP, and 14.8% of those reporting heightened PP. This reinforces our assumption that physicians attempt to avoid prescribing medications to older adults presenting functional decline.

It was notable that the physical status of one tenth of the geriatric inpatients was not assessed before discharge. The number who were not assessed for malnutrition risk was considerably higher (n = 38,855; 72.4%). Given that of the 27.6% assessed, 22.3% presented a moderate to high risk of malnutrition, we can assume that a considerable number of at-risk geriatric inpatients were somehow not assessed. Despite this limitation, we found that malnutrition increased the risk of being polymedicated (OR = 1.411; 95%CI 1.263–1.577; *p* < 0.001), complementing a study by Jyrkkä and Enlund [26] revealing that medication may contribute to poor nutritional status by causing a loss of appetite, gastrointestinal problems, and other alterations in body function. This deserves reflection, considering the well-known association between malnutrition and adverse health outcomes. A study of underweight patients in Switzerland by Tröster and Thalmann [52] pointed to almost a five-fold higher (OR = 4.94, 95%CI 1.11–22.01) risk of institutionalization after discharge. Furthermore, Griffin and O’Neill [56] reported that malnourished older adults were more at risk of hospital readmission, nursing home admission, reduced quality of life, and functional decline. In contrast, our results revealed that people with swallowing disorders (OR = 0.702; 95%CI 0.574–0.858; *p* < 0.001) and eating impairments (OR = 0.714; 95%CI 0.619–0.823; *p* < 0.001) were less at risk of being polymedicated. This might be explained by their difficulties taking solid oral dosage forms, compromising medication adherence [57].

Even though acute pain was assessed more systematically (n = 47,887; 89.2%) than malnutrition risk (n = 14,835; 27.6%), pain management was not optimal. Although only 10.2% (n = 5492) of the geriatric inpatients reported suffering from chronic pain for more than three months, one third (n = 17,895; 33.3%) reported acute pain at hospital discharge. A greater (and statistically significant) proportion of polymedicated inpatients reported acute pain than did non-polymedicated patients (38% vs. 35.8%), whereas the reports of chronic pain were similar (*p* = 0.153). These rates indicate deficient pain medication management in both groups, which is of significant concern considering the well-known functional and mental impacts of pain on older adults, as well as the great healthcare costs [58].

Non-polymedicated inpatients showed greater cognitive impairment in all of the six cognitive variables considered. Moreover, the patients with severe cognitive impairments had fewer medications at hospital discharge. Figueiredo and Teixeira [27] revealed that cognitive impairment was a predictor of noncompliance and difficulty in medication intake, thus increasing the risk of MRPs. However, this pattern contrasted with findings by de Lima and Teixeira [59], in a middle-income country, showing that older adults with mental disorders had greater odds for PP (OR = 22.00, 95%CI 6.11–79.11 for Alzheimer’s disease; OR = 14.73, 95%CI 3.69–58.75 for major depressive disorder; and OR = 10.31, 95%CI 2.44–43.59 for mild cognitive impairment). This difference may be explained by the current concerns of clinicians in high-income countries about potentially inappropriate prescribing and deprescribing practices [54], probably leading them to follow the recommendations of good clinical practice in medication prescription more closely with geriatric populations presenting with cognitive disorders [60]. It may also be that current recommendations on deprescribing potentially futile medications in patients over 65 years old and with a limited life expectancy have shifted clinicians’ focus away from disease prevention and towards maximizing the patient’s quality of life [61]. Furthermore, we noticed that most patients whose cognitive status could not be assessed were non-polymedicated. Although our study could not explain this difference directly, our findings suggested that polymedicated geriatric inpatients tended to have their cognitive function assessed more often.

Geriatric inpatients are susceptible to greater functional decline and adverse health events which are not directly related to the illness for which they were hospitalized [11,14,15]. Additionally, they are at risk of not being able to return to their homes [52]. Therefore, planning a multi-dimensional, patient-centered treatment program as soon as they are hospitalized seems to be crucial to prevent functional decline while it is still reversible [15,62]. The failure to do this may explain why 5% of the older adult hospitalizations in our study ended in death. Our hospital register, from a regional hospital in French-speaking Switzerland, came from a healthcare system that is highly focused on hospital care. Some studies have shown that healthcare systems focused on primary care enable earlier responses to the complex, multi-factorial, and often accumulated needs of geriatric patients [63,64].

Considering that advanced age and PP are the main risk factors for MRPs [24], and that MRPs have a major impact on functional outcomes [21,22], the number of medications that geriatric inpatients have to manage, more or less autonomously, after hospital discharge deserves more attention from the healthcare professionals involved in discharge planning [33].

### 4.1. Study Strengths and Weaknesses

Few studies have descriptively analyzed population-based hospital registers to explore and compare the functional status of polymedicated and non-polymedicated inpatients at hospital discharge. This paper’s in-depth description of the functional status of these populations is a timely addition to the burgeoning body of evidence offering new perspectives on the profiles of polymedicated geriatric inpatients at hospital discharge. Polypharmacy has become a determining factor in some healthcare decisions, notably whether older adults should receive home care services at hospital discharge [65]. However, our finding that non-polymedicated inpatients actually had proportionally more physical and cognitive impairments raises further interesting issues for individual discharge planning.

Our study had some limitations, particularly related to the dataset. In the absence of a unique, encrypted patient identifier, we were unable to track the inpatients over time to detect multiple hospitalizations from 2015–2017. Furthermore, no electronic patient records were available for the adult psychiatry department during that period. Because patients’ medication datasets at admission and during hospitalization are often incomplete or unknown, we were unable to explore changes in the number of prescriptions during their hospitalization. It would be interesting to explore whether hospitalization tends to increase or decrease the number of medications prescribed to geriatric inpatients. Moreover, it would be interesting to explore whether hospitalization is the appropriate period in which to review prescribed medications, reduce inappropriate prescribing, and potentially deprescribe medications across transitions of care in a straightforward, effective way. This could constitute a valuable preventive strategy for the avoidance of MRPs [66].

Another limitation concerned the missing values for some specific variables—such as fall risk and malnutrition risk—which restricted the power of some of the statistical analyses. These missing values revealed gaps in comprehensive geriatric assessment, and in the systematic, structured and rigorous screening of the shared risk factors for geriatric syndromes and frailty that are responsible for adverse health outcomes among older adults—as documented in Inouye’s unifying conceptual model [13]. These gaps and their adverse impacts on geriatric inpatients, healthcare professionals and the healthcare system were reported by Gronewold et al. [67]. Some of our other variables could not be assessed (e.g., gait and balance disorders). Given that walking speed is the strongest determinant of frailty [68], these missing values indicate that the systematic evaluation of frailty in acute care is embryonic. Because hospitalization among older adults is often a precipitating factor for worsening functional and clinical status [11], frailty should be systematically assessed at different stages of hospitalization. The essential components of a comprehensive geriatric assessment should be integrated into daily clinical practice, and should be provided to frail geriatric inpatients in a timely manner in order to minimize the adverse consequences of frailty [69].

### 4.2. Unanswered Questions and Future Research

Due to their complexity, the classes of medications taken at discharge will be analyzed in a separate study describing the medication profiles of geriatric inpatients.

It would be relevant for future research to explore registries from geriatric psychiatry departments, completing the profiles described. It would also be relevant to calculate the prevalence and level of frailty from the available registry variables, and to carry out a more in-depth comparison of the level of frailty among polymedicated and non-polymedicated geriatric inpatients on admission and at discharge. Comparing prescribed medications at admission with those at hospital discharge could facilitate an understanding of the clinical/medication/treatment pathways of polymedicated and non-polymedicated geriatric inpatients.

## 5. Conclusions

Our findings showed that polymedicated geriatric inpatients had proportionally more impairments in general mobility and gait, dependence in lower body care and fatigue, and they presented with a higher malnutrition risk. However, non-polymedicated geriatric inpatients had a proportionally higher number of physical and cognitive impairments.

Comparing the functional status of polymedicated and non-polymedicated patients over 65 years old at hospital discharge is an important means of identifying and monitoring those who are most vulnerable to functional decline. Interventions aimed at preventing functional decline must consider the diversity of factors linking polypharmacy and functional status. Only the systematic in-depth assessment of the functional status of geriatric inpatients will enable the appropriate design of targeted strategies for the prevention of functional impairment and related adverse health outcomes.

## Figures and Tables

**Table 1:** Sociodemographic characteristics of the geriatric inpatients aged 65 or more (n = 53,690), and a comparison between those who were polymedicated (n = 36,266) and non-polymedicated (n = 17,424).

Variables	Geriatric Inpatients Aged 65 or More (n = 53,690)			
	Total n (%) 53,690	Polymedicated 36,266 (67.5)	Non-Polymedicated 17,424 (32.5)	*p*-Value
Sex				0.763 ^a^
Men	25,672 (47.8)	17,357 (67.6)	8315 (32.4)	
Women	28,018 (52.2)	18,909 (67.5)	9109 (32.5)	
Age (years)				
Mean (SD)	78.37 (7.9)	78.75 (7.7)	77.58 (8.3)	<0.001 ^b^
Min–Max	65–106	65–106	65–106	
Med (IQR-75)	78.00 (72–84)	79.00 (72–84)	77.00 (70–84)	
65–74	18,882 (35.2)	11,753 (62.2)	7129 (37.8)	<0.001 ^a^
75–84	21,818 (40.6)	15,485 (71.0)	6333 (29.0)	<0.001 ^a^
85 or more	12,990 (24.2)	9028 (69.5)	3962 (30.5)	<0.001 ^a^
Admitted from				
Home	38,324 (71.4)	24,740 (64.6)	13,584 (35.4)	<0.001 ^a^
Other locations ^d^	15,366 (28.6)	11,526 (75.0)	3840 (25.0)	<0.001 ^a^
Discharged to				<0.001 ^a^
Home	33,771 (62.9)	23,747 (70.3)	10,024 (29.7)	
Other locations ^d^	17,273 (32.2)	12,302 (71.2)	4971 (28.8)	
Died in hospital	2646 (4.9)	217 (8.2)	2429 (91.8)	
Length of stay				<0.001 ^c^
(days)				
Mean (SD)	12.1 (14.5)	13.8 (15.1)	8.6 (12.4)	
Min–Max	1–197	1–197	1–192	
Med (IQR-75)	8 (4–15)	10 (5–17)	4 (1–11)	
65–74				<0.001 ^b^
Mean (SD)	10.3 (14.7)	12.4 (16.0)	6.8 (11.4)	
Min–Max	1–197	1–197	1–192	
Med (IQR-75)	6 (13)	8 (4–15)	3 (1–7)	
75–84				<0.001 ^b^
Mean (SD)	12.4 (14.0)	13.9 (14.4)	8.8 (12.2)	
Min–Max	1–194	1–194	1–189	
Med (IQR-75)	9 (13)	10 (1)	5 (10)	
85 or more				<0.001 ^b^
Mean (SD)	14.3 (14.6)	15.5 (14.8)	11.6 (13.9)	
Min–Max	1–192	1–192	1–170	
Med (IQR-75)	11 (14)	12 (18)	8 (15)	

Note. ^a^ Chi-square test; ^b^ ANOVA; ^c^ Wilcoxon–Mann–Whitney; ^d^ other locations: hospitals and nursing homes.

**Table 2:** Distribution and comparison of the physical status of the polymedicated (n = 36,266) and non-polymedicated (n = 17,424) geriatric inpatients at hospital discharge.

Physical Status	Geriatric Inpatients Aged 65 Years Old or More (n = 53,690)			
	Total n (%)	Polymedicated n (%) 36,266 (67.5)	Non-Polymedicated n (%) 17,424 (32.5)	*p*-Value
Mobility (n = 47,996; 89.4%)				<0.001 ^a^
Full ability n (%)	27,444 (51.1)	19,786 (56.8)	7658 (58.1)	
Impairment n (%)	20,552 (38.3)	15,029 (43.2)	5523 (41.9)	
Mobility for changing position (n = 47,997; 89.4%)				<0.001 ^a^
Full ability n (%)	33,660 (62.7)	24,795 (71.2)	8865 (67.3)	
Impairment n (%)	14,337 (26.7)	10,022 (28.8)	4315 (32.7)	
Gait (n = 47,997; 89.4%)				<0.001 ^a^
Normal n (%)	26,229 (48.9)	18,717 (53.8)	7512 (57)	
Disorder n (%)	21,768 (40.6)	16,100 (46.2)	5668 (43)	
Balance disorders (n = 47,996; 89.4%)				0.002 ^a^
Normal n (%)	31,413 (58.5)	22,935 (65.9)	8478 (64.3)	
Disorder n (%)	16,583 (30.9)	11,882 (34.2)	4701 (35.6)	
Fall risk (n = 31,744; 59.1%)				<0.001 ^a^
Mild-to-moderate risk n (%)	27,203 (50.7)	20,989 (87.7)	6214 (79.5)	
Moderate-to-severe risk n (%)	4541 (8.4)	2943 (12.3)	1598 (20.4)	
Fatigue (n = 47,995; 89.4%)				<0.001 ^a^
Normal n (%)	25,357 (47.2)	17,899 (51.4)	7458 (56.6)	
Disorder n (%)	22,638 (42.2)	16,917 (48.6)	5721 (43.4)	
Upper body care (n = 47,972; 89.3%)				<0.001 ^a^
Full ability n (%)	30,411 (56.6)	22,432 (64.5)	7979 (60.6)	
Impairment n (%)	17,561 (32.7)	12,369 (35.5)	5192 (39.4)	
Lower body care (n = 47,972; 89.3%)				<0.001 ^a^
Full ability n (%)	24,399 (45.4)	17,491 (50.3)	6908 (52.4)	
Impairment n (%)	23,573 (43.9)	17,310 (49.7)	6263 (47.6)	
Upper body dressing/undressing (n = 47,971; 89.3%)				<0.001 ^a^
Full ability n (%)	31,861 (59.3)	23,581 (67.8)	8280 (62.9)	
Impairment n (%)	16,110 (30)	11,219 (32.2)	4891 (37.1)	
Lower body dressing/undressing (n = 47,970; 89.3%)				0.005 ^a^
Full ability n (%)	25,682 (47.8)	18,494 (53.1)	7188 (54.6)	
Impairment n (%)	22,288 (41.5)	16,305 (46.9)	5983 (45.4)	
Eating (n = 47,973; 89.4%)				<0.001 ^a^
Full ability n (%)	40,336 (75.1)	30,212 (86.8)	10,124 (76.9)	
Impairment n (%)	7637 (14.2)	4588 (13.2)	3049 (23.1)	
Drinking (n = 47,974; 89.4%)				<0.001 ^a^
Full ability n (%)	42,915 (79.9)	31,995 (91.9)	10,920 (82.9)	
Impairment n (%)	5059 (9.4)	2808 (8.1)	2251 (17.1)	
Swallowing disorders (n = 47,972; 89.3%)				<0.001 ^a^
Normal n (%)	45,750 (85.2)	33,718 (96.9)	12,032 (91.4)	
Disorder n (%)	2222 (4.1)	1083 (3.1)	1139 (8.6)	
Bladder continence (n = 47,960; 89.3%)				<0.001 ^a^
Normal n (%)	32,644 (60.8)	23,837 (68.5)	8807 (66.9)	
Disorder n (%)	15,316 (28.5)	10,956 (31.5)	4360 (33.1)	
Independence in urination (n = 47,957; 89.3%)				<0.001 ^a^
Normal n (%)	34,912 (65.0)	25,751 (74.0)	9161 (69.6)	
Disorder n (%)	13,045 (24.3)	9040 (26.0)	4005 (30.4)	
Bowel continence (n = 47,960; 89.3%)				<0.001 ^a^
Normal n (%)	40,104 (74.7)	29,722 (85.4)	10,382 (78.8)	
Disorder n (%)	7856 (14.6)	5071 (14.6)	2785 (21.2)	
Independence in defecation (n = 47,960; 89.3%)				<0.001 ^a^
Normal n (%)	36,749 (68.4)	27,137 (78.0)	9612 (73.0)	
Disorder n (%)	11,211 (20.9)	7656 (22.0)	3555 (27.0)	
Hearing (n = 47,943; 89.3%)				0.105 ^a^
Normal n (%)	40,876 (76.1)	11,164 (84.8)	29,712 (85.4)	
Disorder n (%)	7067 (13.2)	1996 (15.2)	5071 (14.6)	
Vision (n = 47,943; 89.3%)				0.105 ^a^
Normal n (%)	5820 (10.8)	11,410 (86.7)	30,713 (88.3)	
Disorder n (%)	42,123 (78.5)	1750 (13.3)	4070 (11.7)	
Acute pain (n = 47,887; 89.2%)				<0.001 ^a^
No acute pain n (%)	29,992 (55.9)	21,552 (62.0)	8440 (64.2)	
Pain n (%)	17,895 (33.3)	13,189 (38.0)	4706 (35.8)	
Chronic pain (n = 47,872; 89.2%)				0.153 ^a^
No chronic pain n (%)	42,380 (78.9)	30,703 (88.4)	11,677 (88.9)	
Pain n (%)	5492 (10.2)	4029 (11.6)	1463 (11.1)	
Malnutrition risk (n = 14,835; 27.6%)				<0.001 ^a^
None n (%)	2840 (5.3)	1797 (16.3)	1043 (27.3)	
Increased n (%)	11,995 (22.3)	9219 (83.7)	2776 (72.7)	
Braden risk (n = 47,751; 88.9%)				<0.001 ^a^
Improbable risk n (%)	38,340 (71.4)	28,510 (82.3)	9830 (75.0)	
Increased risk n (%)	9411 (17.5)	6127 (17.7)	3284 (25)	

Note. ^a^ Chi-square test.

**Table 3:** Distribution and comparison of cognitive status among polymedicated (n = 36,266) and non-polymedicated (n = 17,424) geriatric inpatients.

Cognitive Status	Geriatric Inpatients Aged 65 or More (n = 53,690)			
	Total n (%)	Polymedicated n (%) 36,266 (67.5)	Non-Polymedicated n (%) 17,424 (32.5)	*p*-Value
Alertness/consciousness (n = 47,947; 89.3%)				<0.001 ^a^
Normal n (%)	45,779 (85.3)	34,196 (98.3)	11,583 (88.0)	
Disorder n (%)	2168 (4.0)	591 (1.7)	1577 (12.0)	
Orientation space, time, people (n = 47,949; 89.3%)				<0.001 ^a^
Normal n (%)	37,800 (70.4)	28,102 (80.8)	9698 (73.7)	
Disorder n (%)	10,149 (18.9)	6686 (19.2)	3463 (26.3)	
Concentration (n = 47,948; 89.3%)				<0.001 ^a^
Normal n (%)	42,105 (78.4)	31,377 (90.2)	10,728 (81.5)	
Disorder n (%)	5843 (10.9)	3410 (9.8)	2433 (18.5)	
Verbal expression (n = 47,944; 89.3%)				<0.001 ^a^
Normal n (%)	43,572 (81.2)	32.391 (93.1)	11,181 (85.0)	
Disorder n (%)	4372 (8.1)	2392 (6.9)	1980 (15.0)	
Capacity and skills to react to the demands of daily life (n = 47,948; 89.3%)				<0.001 ^a^
Normal n (%)	34,644 (64.5)	25,493 (73.3)	9151 (69.5)	
Disorder n (%)	13,304 (24.8)	9294 (26.8)	4010 (30.5)	
Ability to learn (n = 47,949; 89.3%)				<0.001 ^a^
Normal n (%)	34,125 (63.6)	25,113 (72.2)	9012 (68.5)	
Disorder n (%)	13,824 (25.7)	9675 (27.8)	4149 (31.5)	

Note. ^a^ Chi-square test.

**Table 4:** Associations between polypharmacy and physical status among geriatric inpatients at discharge (n = 53,690).

Physical Status	Polymedicated/Non-Polymedicated Geriatric Inpatients at Discharge (n = 53,690)	
	Cramér’s V	Sign.
Chronic pain	0.007	<0.153
Hearing	0.007	0.105
Mobility	0.011	0.012
Lower body dressing/undressing	0.013	0.005
Balance disorders	0.014	0.002
Bladder continence	0.016	0.001
Pain assessment	0.020	<0.001
Lower body care	0.020	<0.001
Vision	0.022	<0.001
Gait	0.029	<0.001
Upper body care	0.036	<0.001
Mobility for changing position	0.039	<0.001
Independence in urination	0.044	<0.001
Upper body dressing/undressing	0.046	<0.001
Fatigue	0.046	<0.001
Independence in defecation	0.053	<0.001
Bowel continence	0.079	<0.001
Braden risk	0.082	<0.001
Fall risk	0.100	<0.001
Swallowing disorders	0.118	<0.001
Eating	0.122	<0.001
Malnutrition risk	0.122	<0.001
Drinking	0.131	<0.001

**Table 5:** Associations between PP and cognitive status among geriatric inpatients at discharge (n = 53,690).

Cognitive Status	Polymedicated/Non-Polymedicated Geriatric Inpatients at Discharge (n = 53,690)	
	Cramér’s V	Sign.
Ability to learn	0.037	<0.001
Capacity and skills to react to the demands of daily life	0.037	<0.001
Orientation in space, time, and among people	0.077	<0.001
Concentration	0.118	<0.001
Verbal expression	0.127	<0.001
Alertness/consciousness	0.221	<0.001

**Table 6:** Multivariate logistic regressions predicting PP among discharged geriatric inpatients, adjusted for age, sex and length of stay (LOS; n = 53,690).

Variables	B ^a^	Std. Error	Wald ^b^	Df ^c^	Sig. ^d^	Exp(B) ^e^	95% Confidence Interval for Exp(B)	
							Under Limit	Upper Limit
Constant	1.954	0.278	49.334	1	<0.000	7.058	-	-
Fall risk	−0.161	0.068	5.555	1	0.018	0.851	0.745	0.973
Swallowing disorders	−0.354	0.102	11.936	1	0.001	0.702	0.574	0.858
Malnutrition risk	0.344	0.057	36.989	1	<0.000	1.411	1.263	1.577
Drinking	−0.045	0.086	0.276	1	0.599	0.956	0.807	1.131
Eating	−0.337	0.073	21.563	1	<0.000	0.714	0.619	0.823
Concentration	−0.103	0.071	2.083	1	0.149	0.903	0.785	1.037
Verbal expression	−0.230	0.084	7.558	1	0.006	0.794	0.674	0.936
Alertness/consciousness	−1.669	0.113	219.883	1	<0.000	0.188	0.151	0.235
Sex	−0.004	0.048	0.005	1	0.941	0.996	0.907	1.095
Age	−0.013	0.003	16.319	1	<0.000	0.987	0.980	0.993
Length of hospital stay (LOS)	0.018	0.002	88.784	1	<0.000	1.018	1.014	1.022

Note. ^a^ Estimated multinomial logistic regression coefficients for the models. ^b^ The Wald chi-square test tests the null hypothesis that the estimate equals 0. ^c^ Degrees of freedom for each of the variables included in the model. ^d^ *p*-values. ^e^ Odds ratios for the predictors.

## Data Availability

The data are available upon reasonable request.

## Supplementary Materials

The following are available online at https://www.mdpi.com/article/10.3390/geriatrics6030086/s1, Supplementary Table S1: Missing values; Supplementary Table S2: Distribution of diagnoses using the International Statistical Classification of Diseases and Related Health Problems (ICD-10) and medications increasing fall and/or delirium risk among polymedicated and non-polymedicated geriatric inpatients.
