# Supplementary material for: Functional Status among Polymedicated Geriatric Inpatients at Discharge: A Population-Based Hospital Register Analysis
Source: Geriatrics (Basel). 2021 Sep 3;6(3):86. doi: 10.3390/geriatrics6030086 (PMC8482227; doi:10.3390/geriatrics6030086)
Supplement: Supplementary file 1 [file geriatrics-06-00086-s001.zip › geriatrics-1344823-supplementary.pdf]

geriatrics

Table S1. Missing values

	N	Average	Standard deviation	Missing		Number of outliers	
				Effective	Percentage	Low	High
Age	53690	78.37	7.911	0	0.0	0	850
Length of stay	53690	12.26	16.485	0	0.0	0	1640
Medications at discharge	53690	7.08	5.159	0	0.0	0	1474
Comorbidities	53690	5.25	2.25852	0	0.0	0	0
Mobility	47996	0.43	0.495	5694	10.6	0	0
Mobility for changing position	47997	0.30	0.458	5693	10.6	0	0
Gait	47997	2.97	1.916	5693	10.6	0	1967
Fatigue	47995	0.47	0.499	5695	10.6	0	0
Upper body care	47972	0.37	0.482	5718	10.7	0	0
Lower body care	47972	0.49	0.500	5718	10.7	0	0
Upper body dressing/undressing	47971	0.34	0.472	5719	10.7	0	0
Lower body dressing/undressing	47970	0.46	0.499	5720	10.7	0	0
Eating	47973	0.16	0.366	5717	10.6	0	7637
Drinking	47974	0.11	0.307	5716	10.6	0	5059
Independence in urination	47957	0.27	0.445	5733	10.7	0	0
Bladder continence	47960	0.32	0.466	5730	10.7	0	0
Independence in defecation	47960	0.23	0.423	5730	10.7	0	0
Bowel continence	47960	0.16	0.370	5730	10.7	0	7856
Alertness/consciousness	47947	0.05	0.208	5743	10.7	0	2168
Orientation	47949	0.23	0.460	5741	10.7	0	831
Ability to learn	47949	0.29	0.453	5741	10.7	0	0
Concentration	47948	0.13	0.345	5742	10.7	0	5843
Hearing	47943	0.15	0.375	5747	10.7	0	7067
Vision	47943	0.13	0.355	5747	10.7	0	5820
Verbal expression	47944	0.09	0.288	5746	10.7	0	4372

Table S2. Distribution of diagnoses using the International Statistical Classification of Diseases and Related Health Problems (ICD-10) and medications increasing fall and/or delirium risk among polymedicated and non-polymedicated geriatric inpatients.

**ICD-10**	**Older inpatients aged 65 years old or more (N = 53,690)**			
	**Total n (%)**	Polymedicated n (%) 36,266 (67.5)	Non polymedicated n (%) 17,424 (32.5)	*P*-value
**Number of ICD-10 per geriatric inpatient**				
Min–Max	2–9	2–9	2–9	< 0.001[a]
Mean (SD)	5.3 (2.3)	5.7 (2.2)	4.4 (2.2)	
Med [IQR 25–75]	5 [3–7]	6 [4–8]	3 [3–6]	
**Principal ICD-10 diagnosis (Top 10)**				
Neoplasms (C00–D48)	5,771 (10.7)	3,149 (54.6)	2,622 (45.4)	< 0.001[a]
Mental and behavioral disorders (F00–F99)	3,104 (5.8)	2,138 (68.9)	966 (31.1)	< 0.001[a]
Diseases of the nervous system (G00–G99)	1,876 (3.5)	1,168 (62.3)	708 (37.7)	< 0.001[a]
Diseases of the circulatory system (I00–I99)	9,386 (17.5)	6,928 (73.8)	2,458 (26.2)	< 0.001[a]
Diseases of the respiratory system (J00–J99)	4,278 (8.0)	3,211 (75.1)	1,067 (24.9)	< 0.001[a]
Diseases of the digestive system (K00–K93)	4,453 (8.3)	2,949 (66.2)	1,504 (33.8)	< 0.049[a]
Diseases of the musculoskeletal system and connective tissue (M00–M99)	5,071 (9.4)	3,863 (76.2)	1,208 (23.8)	< 0.001[a]
Diseases of the genitourinary system (N00–N99)	3,317 (6.2)	2,134 (64.3)	1,183 (35.7)	< 0.001[a]
Congenital malformations, deformations, and chromosomal abnormalities (Q00–Q99)	3,598 (6.7)	2,101 (58.4)	1,497 (41.6)	< 0.001[a]
Symptoms, signs, and abnormal clinical and laboratory findings, not elsewhere classified (R00–R99)	6,964 (13.0)	4,583 (65.8)	2,381 (34.2)	0.001[a]
**Medication increasing fall and/or delirium risk**				
Prescribed	14,903 (27.8)	11,396 (32.8)	3,507 (26.6)	< 0.001[a]
Not prescribed	33,041 (61.5)	23,388 (67.2)	9,653 (73.4)	< 0.001[a]

[a] Chi-square test [b] Wilcoxon–Mann–Whitney test
